# Identification of bovine, porcine and fish gelatin signatures using chemometrics fuzzy graph method

**DOI:** 10.1038/s41598-021-89358-2

**Published:** 2021-05-07

**Authors:** Nurfarhana Hassan, Tahir Ahmad, Norhidayu M. Zain, Siti Rahmah Awang

**Affiliations:** 1 Department of Mathematical Sciences, Faculty of Science, Universiti Teknologi Malaysia, 81310 Skudai, Johor Malaysia; 2 Islamic Civilization Academy, Faculty of Social Sciences and Humanities, Universiti Teknologi Malaysia, 81310 Skudai, Johor Malaysia; 3 Azman Hashim International Business School, Universiti Teknologi Malaysia, 81310 Skudai, Johor Malaysia

**Keywords:** Applied mathematics, Pure mathematics, Infrared spectroscopy, Cheminformatics

## Abstract

Gelatin is a protein substance that is widely used in food and pharmaceutical industries. Gelatin is mainly derived from bovine and porcine sources. Fish gelatin is becoming alternative source of gelatin due to concern on health issue and religious constraints. Numerous studies for identification of gelatin sources have been reported. In this study, Fourier transform infrared (FTIR) spectroscopy was used in combination with chemometrics fuzzy autocatalytic set (c-FACS) to distinguish between bovine, porcine and fish gelatins. The gelatin spectra at Amide and 1600–1000 cm^−1^ regions were analyzed using c-FACS and the results were compared to principal component analysis (PCA) and linear discriminant analysis (LDA). The results obtained from c-FACS method showed that each bovine, porcine and fish gelatin possessed dominant wavenumbers at 1470–1475 cm^−1^, 1444–1450 cm^−1^ and 1496–1500 cm^−1^ respectively, which represent their unique signatures. Furthermore, a clear distinction for porcine gelatin was observed in coordinated FACS. The c-FACS method is rigor and faster than PCA and LDA in differentiating the gelatin sources. The novel method promises at least another chemometrics method for FTIR related analysis and the possibilities for other applications are endless.

## Introduction

Gelatin is a protein derived from partial hydrolysis of collagen obtained from animal skin and bones. It is widely used in food and pharmaceutical products due to its unique properties involving gelation, emulsification and stabilization. In food industry, gelatin is commonly used as food additive in various desserts and dairy products. It is also utilized in pharmaceutical industry to manufacture capsules and health supplements^1^. Furthermore, the global gelatin market is rising at a high rate and is expected to grow over the coming years due to the increasing of demands from these two industries^2^. Gelatin is extracted mainly from porcine and bovine sources. They are the most widely used sources for gelatin production due to their abundances and availability. Lately, fish is becoming an alternative source for gelatin due to religious constraints and health concerns. The increase in fish gelatin production is also associated with the growing population of Muslim consumers. The Quran has stated that a Muslim must seek and consume only halal products^3^. Hence, it is essential for Muslims to identify and authenticate the raw materials used in gelatin production, particularly, porcine since it is prohibited for Muslim consumptions. Besides, the issue on possible fraud and mislabeling has become a concern among consumers. Thus, further processes in determination of the sources of gelatin are required for authentication purposes.

Numerous methods have been developed to differentiate bovine, porcine and fish gelatins due to the concern on the halal authenticity issue^4^. Some of the methods involve biochemical, chromatography and spectroscopy procedures coupled with chemometrics techniques. Hidaka and Liu^5^ applied pH drop method coupled with principal component analysis (PCA) to differentiate bovine and porcine gelatins. The method was initially developed in response to the bovine spongiform encephalopathy (BSE) disease outbreak. The gelatin samples were analyzed and differentiated based on the induction time and peak concentration in transformation of hydroxyapatite (HAP). The method used was able to identify the differences between bovine and porcine gelatins with the aid of PCA. Venien and Levieux^6^ devised a biochemical method called enzyme-linked immunosorbent assay (ELISA) to identify bovine and porcine gelatins. Two types of ELISA method, namely indirect ELISA and competitive indirect ELISA were employed in their study. The methods used were able to identify the reactivity and sensitivity differences between bovine and porcine gelatins. Another biochemical method, that has been widely used by several other researchers, is polymerase chain reaction (PCR). The PCR is used to identify the gelatin sources by detecting the presence of deoxyribonucleic acid (DNA)^7^. Jannat et al.^8^ applied PCR method to determine DNA of porcine source. The PCR method was able to detect and identify the presence of porcine DNA and other sources of gelatin. In short, the ELISA and PCR were able to confirm the sources of gelatins. However, the procedures are time consuming and require high-end equipment to execute them. Other methods that have commonly been used in determining the sources of gelatin are high-performance liquid chromatography (HPLC) and Fourier transform infrared (FTIR) spectroscopy. Widyaninggar et al.^9^ and Ismail et al.^10^ applied HPLC in combination with principal component analysis (PCA) to identify different sources of gelatin. The PCA score and loading plot from their studies showed that the gelatin sources were clustered into different groups. On the other hand, Hashim et al.^11^ applied PCA and discriminant analysis in combination with FTIR spectroscopy to classify gelatin sources, while Cebi et al.^12^ and Zilhadia et al.^13^ applied PCA in their analyses. These researchers combined chemical methods with chemometrics for their gelatin authentication analyses due to the difficulties in identifying the differences between the sources if they just rely on a single method. The gelatin sources have large similarity of protein structure, hence further analysis using chemometrics method is often required. According to Premanandh and Salem^14^, PCA and linear discriminant analysis (LDA) are the most common chemometrics methods used for chemical data analysis. The methods are usually used for classification of samples due to their capabilities in categorizing the samples into different groups^15^. However, these statistical methods require data pre-processing which often time consuming and tedious^4^. Hence, a robust and rapid chemometrics approach is needed to assist the halal authentication of gelatins purpose.

In this paper, chemometrics fuzzy autocatalytic set (c-FACS) method in form of fuzzy graph is used in combination with FTIR spectroscopy to determine the spectra of bovine, porcine, and fish gelatins. According to Irfanita et al.^16^, the FTIR method is reliable and effective for authentication analysis, due to its ability to offer rapid results and unique spectrum of samples. In addition, FTIR has been widely used in analysis involving gelatin^14^. However, chemometrics method is still needed in most chemical analyses, including FTIR, in order to further differentiate the gelatins by analyzing the obtained chemical dataset. In this study, the spectra obtained from FTIR are analyzed using a new developed chemometrics method called c-FACS. The ordinary FACS was first introduced by Ahmad et al.^17^ to model a waste incineration process in form of a dynamic graph. Later, Ashaari et al.^18^ used FACS to model pressurized water system (PWR). The method used was able to determine dominant variables in their respective systems. In this paper, c-FACS method is used in combination with FTIR spectroscopy to identify the signatures of bovine, porcine and fish gelatins. In addition, an advanced form of FACS, namely, coordinated FACS, is utilized to visualize the emerged pattern of each gelatin spectra in the Euclidean space. The coordinated FACS was first introduced by Bakar et al.^19^ to display further information and connection between the variables. The results obtained from the c-FACS analysis of gelatin are compared to the results obtained from Principal component analysis (PCA) and linear discriminant analysis (LDA).

## Materials and methods

### Sample preparation

Three different gelatin samples of bovine (Sigma-Aldrich, St. Louis, MO, USA), porcine (Sigma-Aldrich, St. Louis, MO, USA) and fish (Sigma-Aldrich, St. Louis, MO, USA) sources were used in this study (see Table 1). The bloom value for fish gelatin in Table 1 is not given by Sigma-Aldrich since Thies^20^ reported that cold-water fish gelatin typically does not gel at 10 °C, hence its bloom strength is not apparent. The samples at different concentrations were dissolved in distilled water using an ultrasonic water bath (Fisher Scientific, Germany) at 45 °C for 20 min until clear solution was acquired for each sample. A total of 81 standard gelatin solutions which consists of 27 samples for each bovine, porcine and fish gelatins of nine concentrations between 4% (w/v) to 20% (w/v) were prepared for this study.

**Table 1:** General descriptions of gelatin samples used in this study.

Gelatin	Company name	Batch no	Gelatin type	Bloom value
Bovine	Sigma-Aldrich	SLBN8199V	B	~ 225
Porcine	Sigma-Aldrich	SLBQ9498V	A	~ 300
Cold water fish	Sigma-Aldrich	SLBQ3114V	Solid	Not stated

### FTIR Instrumentation

Spectrum Two FTIR spectrometer (Perkin Elmer, USA) with attenuated total reflection (ATR) accessory equipped with diamond crystal was used to record spectra of the gelatin samples. They were recorded in a range of 4000–450 cm^−1^ with 4 cm^−1^ resolution for 32 scans. All measurements were performed at room temperature around 25 degree Celsius. A background spectrum was recorded before each measurement. The spectra of the samples were subtracted against the background spectrum and the results were presented in absorbance unit. The FTIR spectra of the 81 gelatins were processed and referred to their baseline using Spectrum 10 software (PerkinElmer, USA). The absorbance data from the spectra were collected for further analysis by the following methods.

### Chemometrics fuzzy autocatalytic set (c-FACS)

The concept of fuzzy autocatalytic set (FACS) was first introduced by Ahmad et al.^17^, as a result of merger between fuzzy graph and autocatalytic set (ACS). Fuzzy graph is a graph that incorporates fuzziness and ACS is a concept introduced by Jain and Krishna^21^ to represent catalytic interactions between variables or molecules in a form of a graph. Ahmad et al.^17^ then implemented the concept of fuzziness in ACS to develop FACS. The formal definition of FACS is given as follows:

#### Definition 1.

FACS is a sub graph where each of whose nodes has at least one incoming link with membership value, $\mu (e_{i} ) \in \left( {0,1} \right], \, \forall e_{i} \in E$.

**Figure 1:**
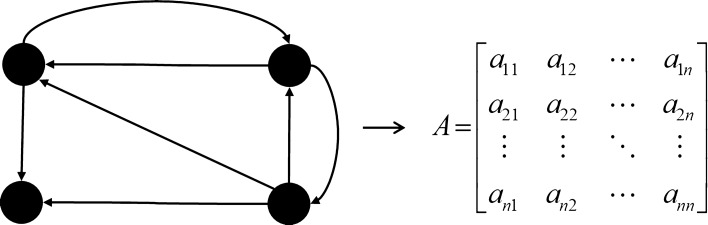
An FACS graph and its adjacency matrix.

The FACS graph can be transformed into a matrix form as shown in Fig. 1. The entries of the matrix are the membership values.

The chemometrics analysis using chemometrics FACS (c-FACS) involves the development of fuzzy graph model of the FTIR spectra of gelatins and follows by the construction of its adjacency matrix. The graph represents the process of absorption of light by the gelatin molecule during FTIR analysis. The graph is then converted into a matrix by determining the membership values. Graph dynamic procedure by Ahmad et al.^17^ is used to identify the dominant matrix. The procedure involves several steps involving the identification of Perron-Frobenius eigenvectors (PFE) of the matrix as follows:

*Step 1 *Determine the adjacency matrix of the $n \times n$ matrix, $C = [c_{ij} ]$, where

$$c_{ij} = \left\{ \begin{array}{ll} \mu (e_{i})&\quad {\text{if }}\,\,(i,j) \in E \hfill \\ \, 0&\quad{\text{ if}}\,\, (i,j)\,\, \notin E \hfill \\ \end{array} \right.$$

*Step 2* Determine the lowest value of ${\text{X}}_{{\text{i}}}$ of PFE and its corresponding set of vertices:

$$V = \left\{ {i \in S\left| {X_{i} = {\text{min}}_{j \in s} } \right.X_{j} ,S = \left\{ {1,2,...,n} \right\}} \right\}$$

*Step 3* The lowest value of ${\text{X}}_{{\text{i}}}$ of PFE and its corresponding set of vertices and edges are discarded. A new graph is formed with *n*-1 vertices.

*Step 4* Construct the adjacency matrix of the new updated graph with size of $(n - 1) \times (n - 1)$.

**Figure 2:**
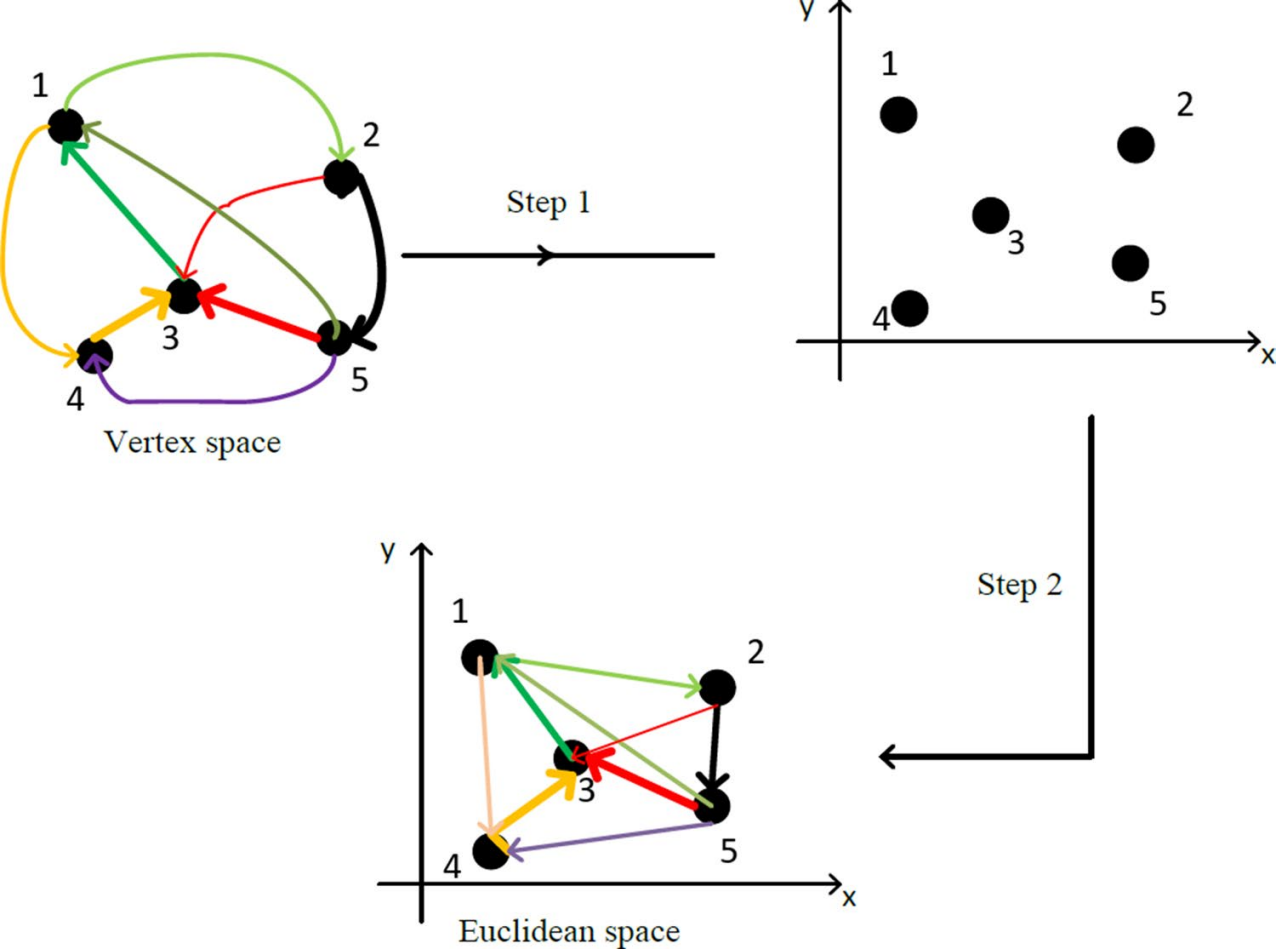
Schematic illustration transformation of FACS with 5 vertices to Euclidean space^22^.

The updated new matrix is called dominant matrix. The dominant matrix indicates the dominant absorbance in association to the wavenumber of FTIR spectra. The difference among each gelatin is observed by determining its respective dominant wavenumbers. Further analysis using coordinated FACS is executed to transform the graph into the Euclidean space in order to determine the pattern and signature of each gelatin spectrum (see Fig. 2).

The transformation is comprised of several procedures to obtain the coordinates of the nodes in Euclidean space. The matrices induced from FACS gelatin spectra are transformed into Laplacian form and followed by execution of procedures introduced by Carmel et al.^23^. Several important properties of FACS and its mathematical structures related to the transformation have been studied by Bakar et al.^19^. The *x-*coordinates of the FACS nodes are obtained by finding the Fiedler vector of the Laplacian matrix. According to Bakar et al.^19^, the idea of obtaining the *x-*coordinates through Fiedler vector is based on the concept of minimization of Tutte-Hall energy function. The Fiedler vector is then obtained by solving the eigenvalue problem of Laplacian. Moreover, the *y-*coordinates of the FACS nodes are determined by using a modified concept of minimization of hierarchy energy function which is equivalent to solving its optimal arrangement^19^. The coordinates of the FACS nodes are then displayed in Euclidean space.

On the other hand, statistical methods of principal component analysis (PCA) and linear discriminant analysis (LDA) were performed to classify the gelatin spectra. The PCA method is one of the most common statistical methods used in analyzing chemical data^4^. The method operates in such a way that the dimension of the data is reduced while retaining the most relevant information. The PCA decomposes the data matrix to components called principal components (PCs) that describe the variations in the data set. The principal components are the linear combinations of the original variables that generate the axes^24^. The results are displayed in form of score and loading plots. The score plot is constructed from the first two principal components that describes the variability of the data set and the loading plot exhibits the relationship among the variables^25^. Another statistical method that is also commonly used for classification analysis is LDA. The LDA is closely related to PCA, since both methods involve with linear transformation of the dataset, but the transformation in LDA is mainly based on finding the linear discriminants that maximize the separation between different classes, while PCA focuses on finding PCs that maximize the variance in the data^26, 27^. In this study, the PCA analysis was carried out using Minitab software (Pennsylvania, USA) while LDA was performed using R software 4.0.4.

## Results and discussion

### FTIR spectra of gelatins

**Figure 3:**
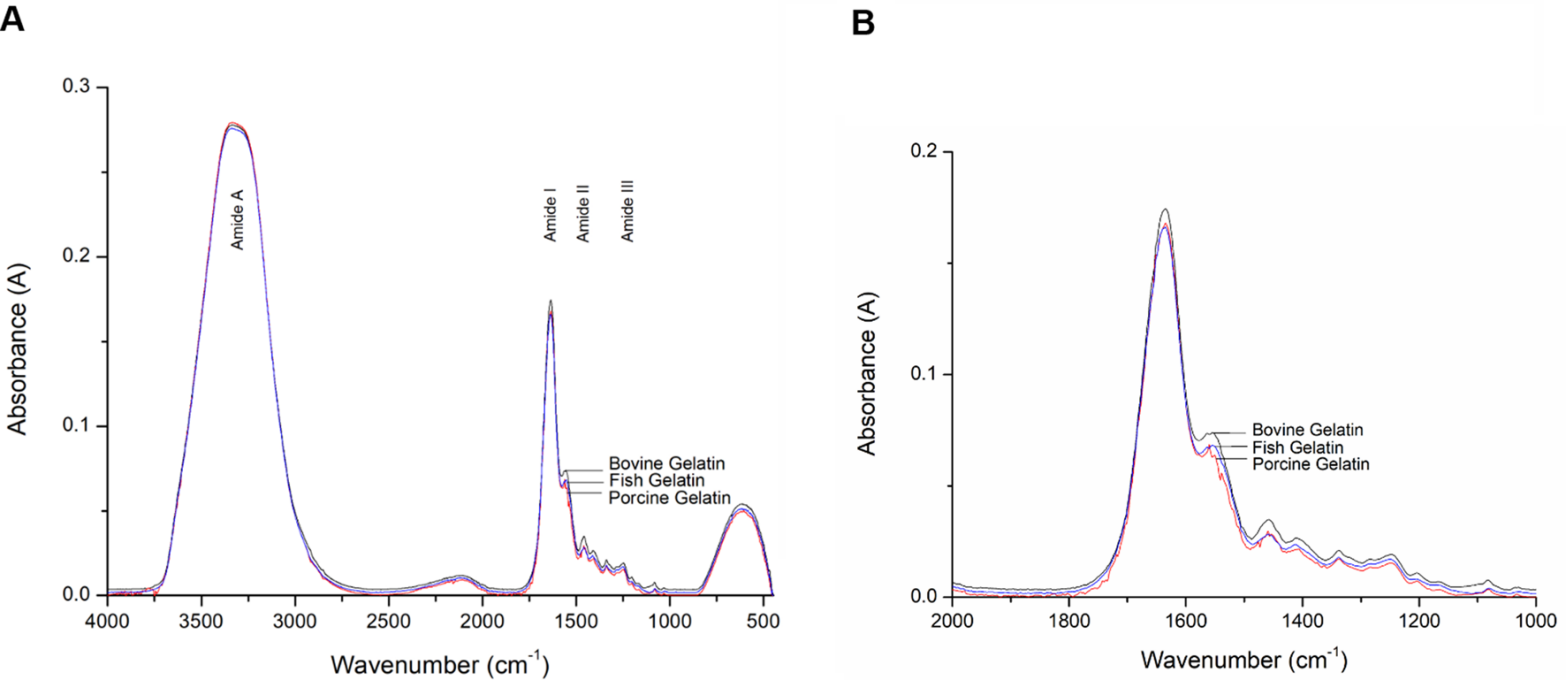
(**A**) Full scale FTIR spectra of bovine, porcine and fish gelatins and (**B**) enlarged spectra of bovine, porcine and fish gelatins at 2000–1,000 cm^-1^.

The spectra of bovine, porcine and fish gelatins from FTIR are shown in Fig. 3. The spectra of these gelatins showed large similarities with major peaks were observed at wavenumbers of 3310–3270 cm^-1^ (Amide A), 1700–1600 cm^-1^ (Amide I), 1550—1400 cm^-1^ (Amide II) and 1240–670 cm^-1^ (Amide III). Similar spectra patterns and peaks were obtained by Hashim et al.^11^ and Zilhadia et al.^13^ in their analysis of bovine and porcine gelatins, while the Amide A and II regions were adopted as suggested by Barth^28^.

At each wavenumber region, there exist different factors that responsible for the infrared absorption. Amide A region is associated with O–H stretch and minor N–H vibration^11^. Smith^29^ stated that the O–H stretch indicates the presence of water while Hashim et al.^11^ and Cebi et al.^12^ reported Amide I region is associated with C=O stretch and bending of N–H bond with minor C–N stretch. Amide II band is caused by deformation of N–H bonds. Amide III absorption depends on C–N stretching vibrations coupled to N–H bend^11^ and Muyonga et al.^30^ claimed that the low intensity band in Amide III region is due to loss of triple helix state during gelatin extraction process.

**Figure 4:**
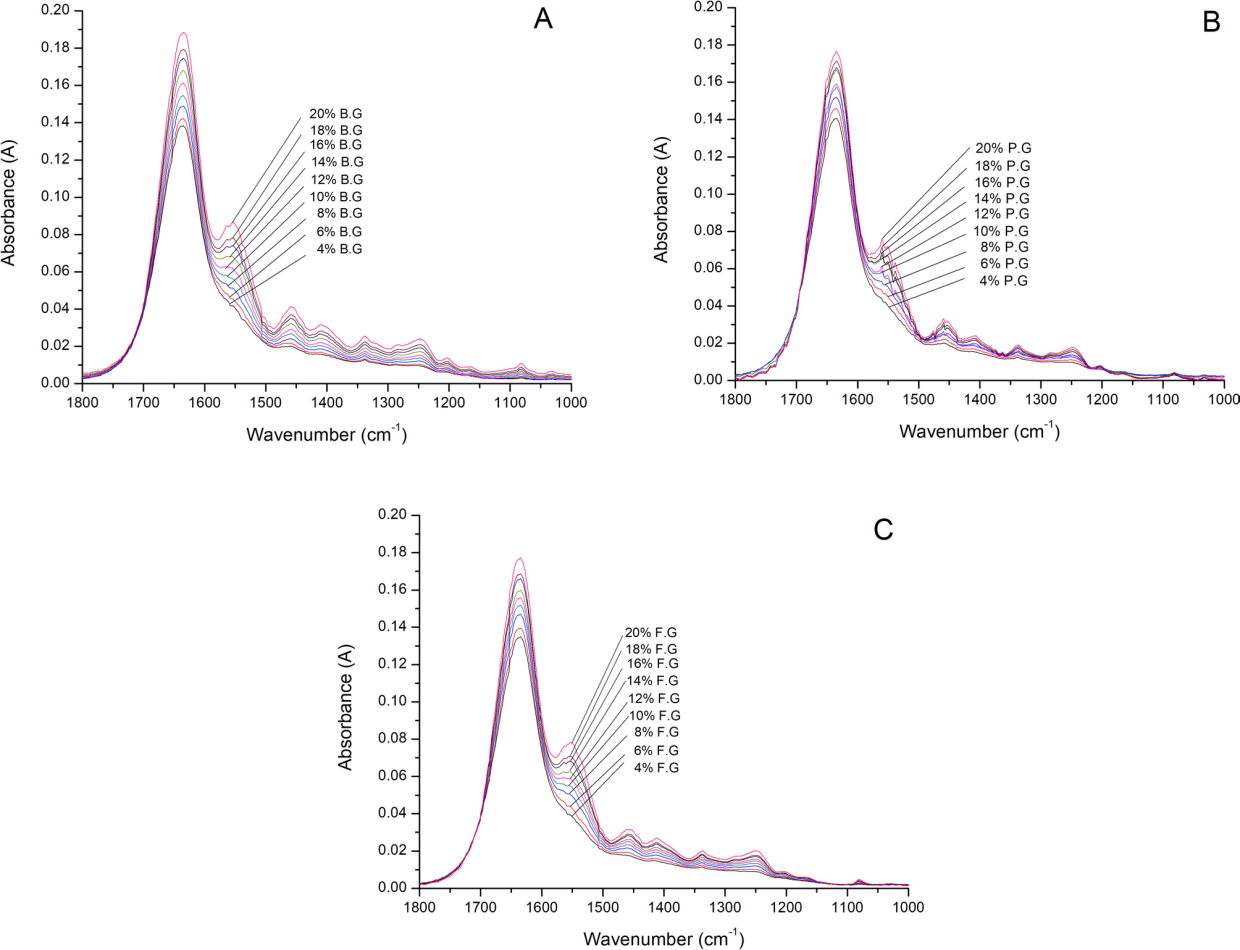
Concentration-dependent FTIR spectra of (**A**) bovine gelatin (B.G), (**B**) porcine gelatin (P.G) and (**C**) fish gelatin (F.G).

The absorption bands in the Amide regions provide some information of gelatin that resulted into wide and overlapping bands of the FTIR spectra. Gelatin samples at different concentrations were analyzed to determine the sensitivity and capability of the FTIR in discriminating the samples at different concentrations. As a result, the FTIR spectra of the gelatins samples at different concentrations are exhibited accordingly (see Fig. 4). The FTIR analysis is seen to be sensitive and capable in discriminating samples with different concentrations.

The next two sections describe the novel chemometrics analysis using chemometrics fuzzy autocatalytic set (c-FACS) and followed by statistical approaches for the FTIR spectra of gelatins.

### C-FACS analysis

After the determination of spectra of bovine, porcine, and fish gelatins using FTIR analysis, the differences between the three gelatins were further identified using chemometrics fuzzy autocatalytic set (c-FACS) technique. The c-FACS technique involves the representation of the FTIR in a form of graph and its matrix, analysis using FACS graph dynamic procedure to determine the dominant matrix and transformation of the FACS graph into coordinated form in Euclidean space. The analysis is performed using Matlab.

**Figure 5:**
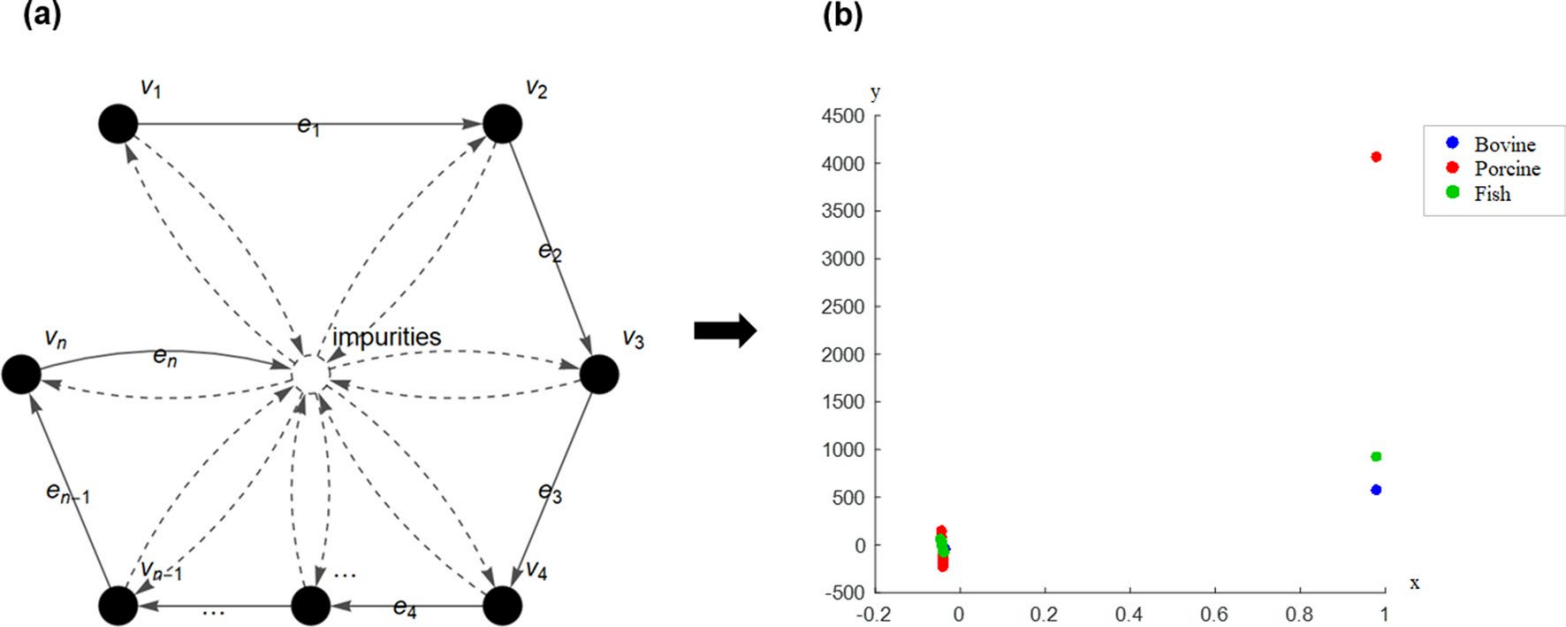
(**a**) FACS graph of FTIR gelatin spectra and (**b**) coordinated FACS of FTIR gelatin spectra.

The FTIR spectra of the gelatins at Amide regions with wavenumbers of 1600–1000 cm^−1^ were transformed into FACS graph (see Fig. 5a) for further identification.

The set of vertices $V = \{ v_{1} ,v_{2} ,v_{3} ,v_{4} , \ldots ,v_{n - 1} ,v_{n} \}$ represents wavenumber and the set of edges $E = \{ e_{1} ,e_{2} ,e_{3} ,e_{4} , \ldots ,e_{n - 1} ,e_{n} \}$ represents transition of the gelatin molecule to the next wavenumber during the emission of light at each region. Absorbance values and possible interference by impurities during the FTIR analysis were described as the membership values of the FACS graph. The FACS graph was converted into a square matrix whereby the entries represent the absorbance data. Then, its dominant matrix was obtained based on the graph dynamic procedure to determine the differences between the gelatins with respect to the wavenumbers. As a result, the dominant wavenumbers for each bovine, porcine and fish gelatin at 1600–1000 cm^−1^, Amide II and Amide III regions were identified, respectively. Table 2 illustrates the dominant output matrix of each gelatin at 1600–1000 cm^−1^ region.

**Table 2:** Dominant output matrices with respective wavenumbers regions for bovine, porcine and fish gelatin spectra in 1600–1000 cm^−1^ region.

Gelatins	Output matrix	Wavenumbers (cm^−1^)
Bovine	$$\left[ \begin{gathered} {0}{\text{.0821 0}}{.0802 0}{\text{.0785 0}}{.0768 0}{\text{.0736}} \hfill \\ {0}{\text{.0590 0}}{.0588 0}{\text{.0587 0}}{.0586 0}{\text{.0584}} \hfill \\ {0}{\text{.0549 0}}{.0545 0}{\text{.0540 0}}{.0537 0}{\text{.0531}} \hfill \\ {0}{\text{.0399 0}}{.0393 0}{\text{.0387 0}}{.0379 0}{\text{.0360}} \hfill \\ {0}{\text{.0247 0}}{.0250 0}{\text{.0253 0}}{.0254 0}{\text{.0255}} \hfill \\ \end{gathered} \right]$$	$$\left[ \begin{gathered} {1600 1599 1598 1597 1595} \hfill \\ {1575 1574 1573 1572 1570} \hfill \\ {1550 1549 1548 1547 1545} \hfill \\ {1525 1524 1523 1522 1520} \hfill \\ {1475 1474 1473 1472 1470} \hfill \\ \end{gathered} \right]$$
Porcine	$$\left[ \begin{gathered} {0}{\text{.0829 0}}{.0809 0}{\text{.0790 0}}{.0773 0}{\text{.0723}} \hfill \\ {0}{\text{.0577 0}}{.0575 0}{\text{.0574 0}}{.0574 0}{\text{.0574}} \hfill \\ {0}{\text{.0546 0}}{.0540 0}{\text{.0532 0}}{.0524 0}{\text{.0492}} \hfill \\ {0}{\text{.0379 0}}{.0372 0}{\text{.0365 0}}{.0356 0}{\text{.0347}} \hfill \\ {0}{\text{.0248 0}}{.0245 0}{\text{.0242 0}}{.0239 0}{\text{.0231}} \hfill \\ \end{gathered} \right]$$	$$\left[ \begin{gathered} {1600 1599 1598 1597 1594} \hfill \\ {1575 1574 1573 1572 1569} \hfill \\ {1550 1549 1548 1547 1544} \hfill \\ {1525 1524 1523 1522 1519} \hfill \\ {1450 1449 1448 1447 1444} \hfill \\ \end{gathered} \right]$$
Fish	$$\left[ \begin{gathered} {0}{\text{.0808 0}}{.0790 0}{\text{.0773 0}}{.0756 0}{\text{.0739}} \hfill \\ {0}{\text{.0563 0}}{.0562 0}{\text{.0561 0}}{.0560 0}{\text{.0560}} \hfill \\ {0}{\text{.0539 0}}{.0535 0}{\text{.0531 0}}{.0527 0}{\text{.0524}} \hfill \\ {0}{\text{.0398 0}}{.0391 0}{\text{.0384 0}}{.0375 0}{\text{.0365}} \hfill \\ {0}{\text{.0246 0}}{.0242 0}{\text{.0238 0}}{.0234 0}{\text{.0230}} \hfill \\ \end{gathered} \right]$$	$$\left[ \begin{gathered} {1600 1599 1598 1597 1596} \hfill \\ {1575 1574 1573 1572 1571} \hfill \\ {1550 1549 1548 1547 1546} \hfill \\ {1525 1524 1523 1522 1521} \hfill \\ {1500 1499 1498 1497 1496} \hfill \\ \end{gathered} \right]$$

The dominant wavenumbers for bovine were identified at 1470–1475 cm^−1^, while the dominant wavenumbers for porcine were detected at 1444–1450 cm^−1^ and the dominant wavenumbers for fish were observed at 1496–1500 cm^−1^ with respect to 1600–1000 cm^−1^ region. For Amide II region, the dominant wavenumbers for bovine, porcine and fish gelatins were identified at 1473–1480 cm^−1^, 1441–1448 cm^−1^ and 1490–1496 cm^−1^, respectively. At Amide III region, the dominant wavenumbers for bovine were identified at 786–810 cm^−1^, 1228–1232 cm^−1^ and 1249–1252 cm^−1^, while dominant wavenumbers for porcine were observed at 678–680 cm^−1^, 1071–1077 cm^−1^ and 1087–1096 cm^−1^, and as for fish, the dominant wavenumbers were detected at 837–844 cm^−1^, 853–862 cm^−1^ and 1280–1303 cm^−1^. These dominant wavenumbers were determined based on the output matrix of the c-FACS analysis. The dominant wavenumbers obtained for bovine gelatin at 1470–1475 cm^−1^, porcine gelatin at 1444–1450 cm^−1^ and fish gelatin at 1496–1500 cm^−1^ are indication of their unique respective signatures when c-FACS is executed.

Further analysis using coordinated FACS was performed to analyze the difference of the gelatins in 2D Euclidean space. The transformation procedure of the FACS graph of FTIR gelatin spectra into the coordinated FACS was adopted as outlined in Bakar et al.^19^. The *x* and *y* coordinates of the FACS graph are determined by identifying the Fiedler vector of the Laplacian matrix and solving optimal arrangement y^*^ using Conjugate Gradient method, respectively. The coordinated FACS of gelatin spectra at 1600–1000 cm^−1^ region is shown in Fig. 5b. The coordinated FACS exhibits distinct patterns for bovine, porcine and fish gelatin samples. The blue, red and green nodes represent bovine, porcine and fish gelatins, respectively. Based on the figure, each gelatin sample exhibited a slightly different pattern and their nodes are dispersed at different locations, particularly in the right area of the graph**.** The three types of gelatin were discriminated accordingly, especially porcine which was clearly separated from bovine and fish gelatin. Porcine gelatin is located distinctly and at higher location while fish gelatin is positioned slightly above bovine. This result showed that porcine gelatin has a very unique characteristic compared to the others. The c-FACS was able to identify the differences between bovine, porcine and fish gelatins based on the dominant wavenumbers and location of the gelatin nodes in the Euclidean space.

### Chemometrics using statistical techniques

**Figure 6:**
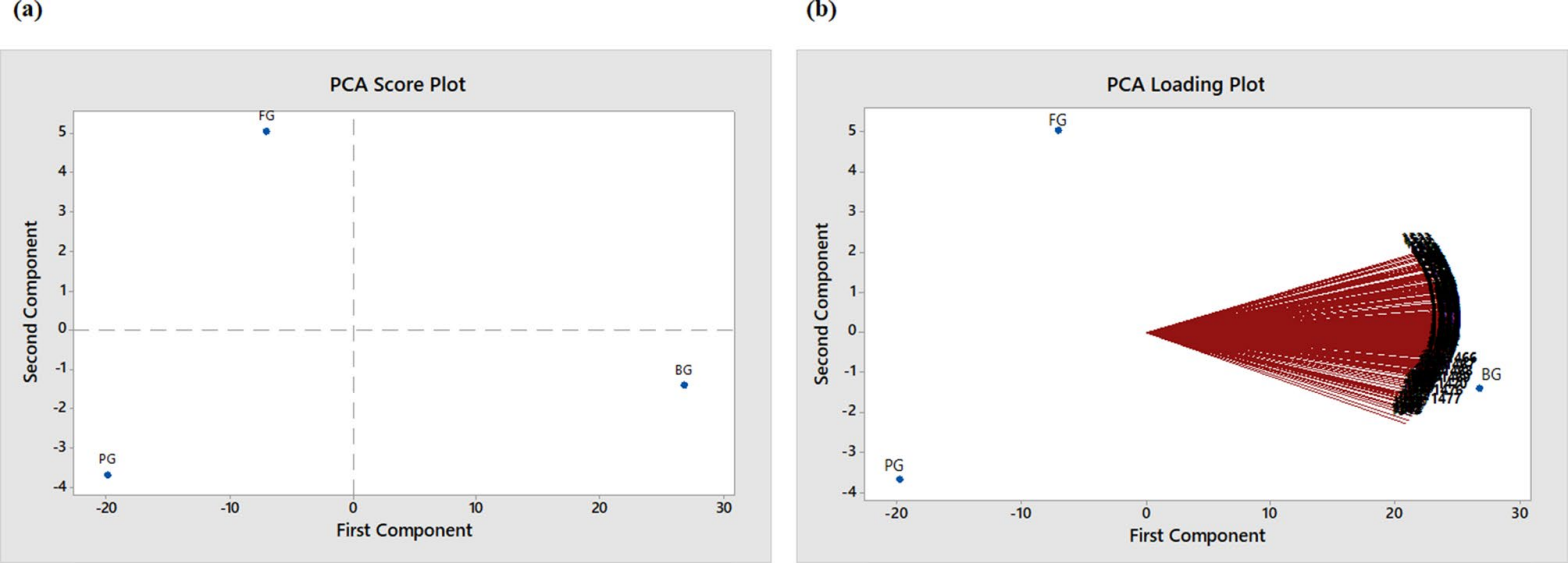
PCA (**a**) score plot and (**b**) loading plot for bovine (BG), porcine (PG) and fish (FG) gelatins.

**Figure 7:**
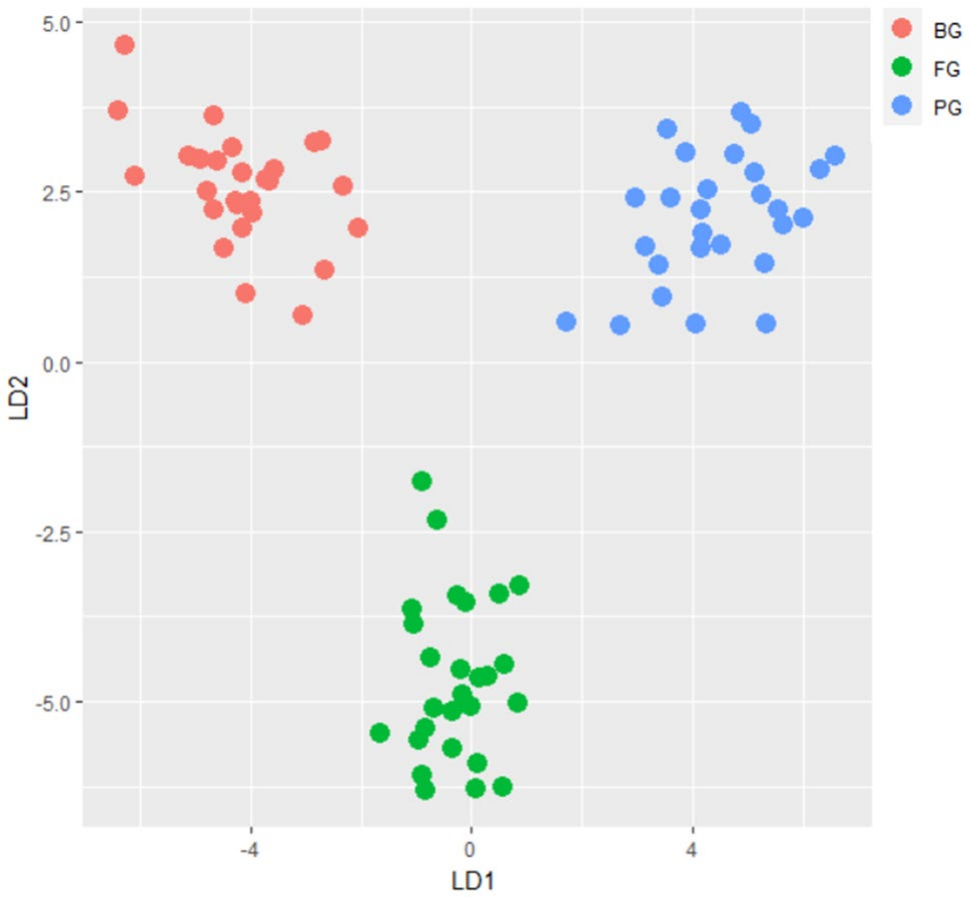
LDA plot for bovine (BG), porcine (PG) and fish (FG) gelatins.

Principal component analysis (PCA) and linear discriminant analysis (LDA) are statistical-based chemometrics methods that are commonly used for classification of samples. The PCA reduces the dimension of variables by constructing principal components (PCs), while LDA maximizes the separation between classes by finding linear discriminant function, which is a linear combination of a set of variables. According to Livingstone^31^, the scores for the first two PCs in PCA analysis are sufficient in describing the information of the data set. The scores of the PCs are displayed in score plot and the correlation between their components and variables are demonstrated in loading plot. On the other hand, LDA plot displays results into groups. The PCA is performed at every concentration to determine the classifications and significant wavenumbers of the gelatins concentrations and compare them with c-FACS, while LDA was able to yield samples classifications only. Minitab and R software are used to compute the PCA and LDA of the gelatins, respectively. The spectral range of 1600–1000 cm^−1^ is expected to provide significant information of the spectra as reported by Smith^29^. The PCA’s score and loading plots are displayed in Fig. 6 and LDA plot is exhibited in Fig. 7.

The clusters of the gelatin samples with respect to its two PC’s components are displayed in the score plot (see Fig. 6a) and LDA plot with respect to its two discriminant functions is illustrated in Fig. 7. Both PCA and LDA shows that bovine, porcine and fish gelatin are clearly separated and classified into different groups. The PCA loading plot shows the correspond wavenumbers for each gelatin (see Fig. 6b) whereby the wavenumbers at 1600–1000 cm^−1^ region are observed to be highly correlated to bovine gelatin. Furthermore, the most significant and closest wavenumbers to the bovine sample are identified in the range of 1466–1477 cm^−1^. Almost similar readings (1470–1475 cm^−1^) are recorded when c-FACS is employed. However, PCA failed to determine the significant wavenumbers that correlated to porcine and fish gelatins, while LDA was able to classify them into groups. Additionally, the computing time for c-FACS (9.5 s) is faster than PCA (28.24 s) and LDA (1.47917 min) with respect to data set for 1600–1000 cm^−1^ region.

### C-FACS analysis on published data

**Figure 8:**
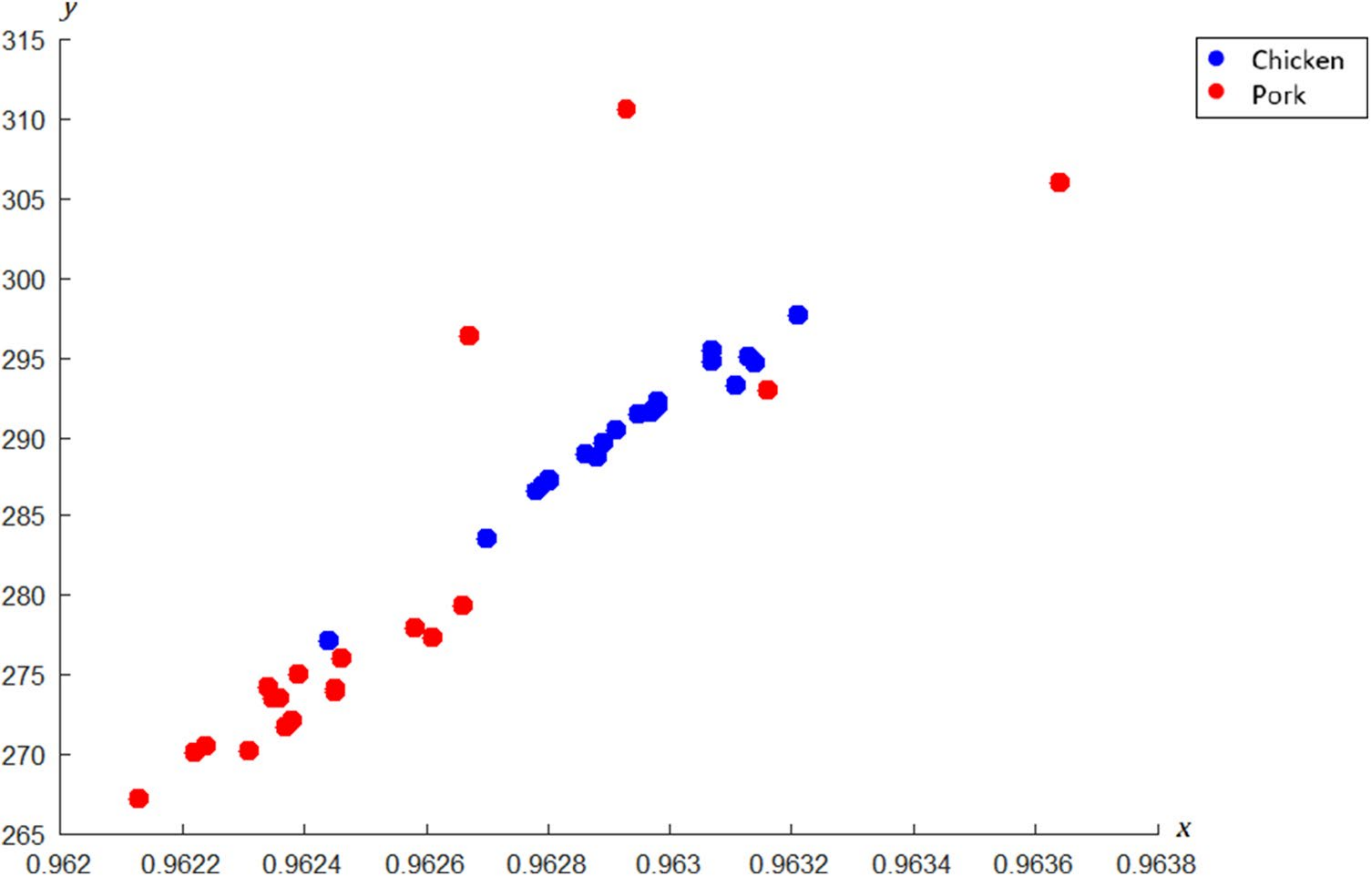
Coordinated c-FACS of chicken and pork meats.

The c-FACS is performed on published data of meat products to validate the technique. The data of chicken and pork meats are obtained from Al-Jowder et al.^32^. In their study, the FTIR spectra of the meat products were analyzed using PCA and clear distinction of the meats were observed. In this paper, the FTIR spectra are analyzed using c-FACS and the results are compared against PCA. The dominant wavenumbers and coordinated c-FACS are determined and presented in Table 3 and Fig. 8, respectively.

**Table 3:** Dominant wavenumbers for chicken and pork meats.

Meat	Wavenumbers (cm^−1^)
Chicken	$$\left[ \begin{gathered} 1418.3\;1416.3\;1414.4\;1408.6 \hfill \\ 1522.5\;1520.5\;1518.6\;1512.8 \hfill \\ 1557.2\;1555.3\;1553.3\;1547.6 \hfill \\ 1591.9\;1590.0\;1588.1\;1582.3 \hfill \\ \end{gathered} \right]$$
Pork	$$\left[ \begin{gathered} 1071.0\;1069.0\;1067.1\;1042.0 \hfill \\ 1522.5\;1520.5\;1518.6\;1493.5 \hfill \\ 1557.2\;1555.3\;1553.3\;1528.3 \hfill \\ 1591.9\;1590.0\;1588.1\;1563.0 \hfill \\ \end{gathered} \right]$$

**Figure 9:**
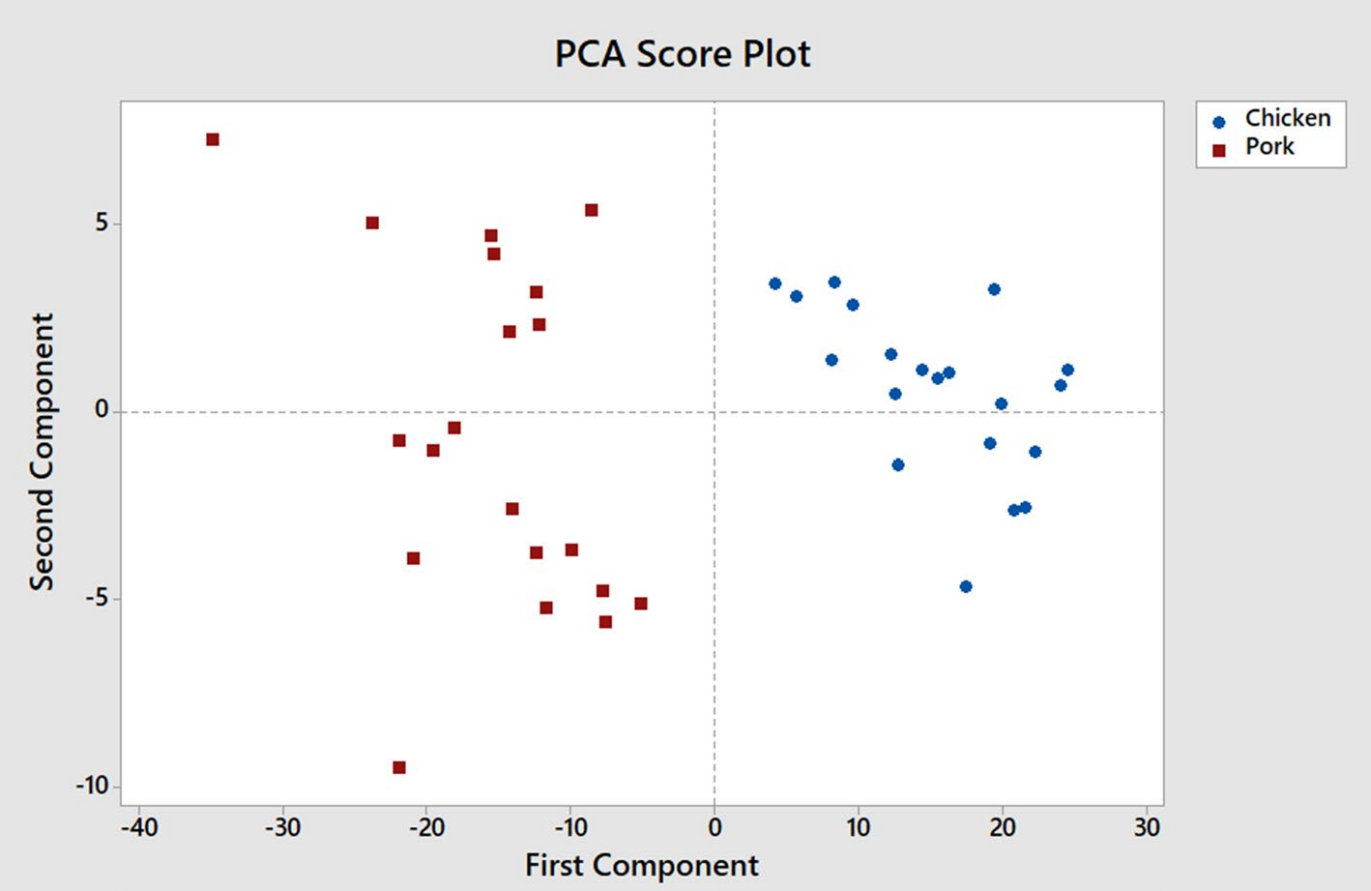
PCA plot of chicken and pork meats.

The results show that both meats have unique and distinct signatures and patterns. The chicken’s dominant wavenumbers are at 1408.6–1418.3 cm^−1^ region and pork’s dominant wavenumbers are at 1042.0–1071.0 cm^−1^. Interestingly, the dominant wavenumber of pork meat at 1071.0 cm^−1^ is similar to the dominant wavenumber of porcine gelatin identified earlier. Thus, the observed range may signify the signature of pig derivatives. In addition, the patterns of both meats in coordinated c-FACS have shown that chicken and pork are clustered at different locations. Similar cluster result was obtained using PCA (see Fig. 9) and by Al-Jowder et al.^32^.

## Conclusion

A chemometrics method using fuzzy graph approach, namely, chemometrics fuzzy autocatalytic set (c-FACS) is introduced to identify and differentiate gelatin sources. The signature wavenumbers for bovine, porcine and fish gelatins were identified, which signify their unique properties. In addition, the nodes of the non-halal porcine gelatin in c-FACS plot displayed distinct pattern compared to the others. The distinct pattern and signature wavenumbers obtained for each gelatin signify their differences and unique characteristics. Furthermore, the c-FACS outperforms PCA and LDA in computing time. Hence, c-FACS offers a new rigor chemometrics method in identification of sources of gelatin, particularly, for halal authentication purposes. The c-FACS promises at least another chemometrics method for FTIR related analysis and the possibilities for other applications are endless.
